# Different Heavy Metal Accumulation Strategies of Epilithic Lichens Colonising Artificial Post-Smelting Wastes

**DOI:** 10.1007/s00244-015-0180-5

**Published:** 2015-07-09

**Authors:** Kaja Rola, Piotr Osyczka, Alina Kafel

**Affiliations:** Department of Plant Taxonomy, Phytogeography and Herbarium, Institute of Botany, Jagiellonian University, Kopernika 27, 31-501 Kraków, Poland; Department of Polar Research and Documentation, Institute of Botany, Jagiellonian University, Kopernika 27, 31-501 Kraków, Poland; Department of Ecotoxicology and Animal Physiology, Faculty of Biology and Environmental Protection, University of Silesia, Bankowa 9, 40-007 Katowice, Poland

## Abstract

Lichens appear to be essential and effective colonisers of bare substrates including the extremely contaminated wastes of slag dumps. This study examines the metal accumulation capacity of epilithic lichens growing directly on the surface of artificial slag sinters. Four species representing different growth forms, i.e., crustose *Candelariella aurella*, *Lecanora muralis*, and *Lecidea fuscoatra* and fruticose *Stereocaulon nanodes*, were selected to evaluate the relationships between zinc, lead, cadmium, and nickel contents in their thalli and host substrates. Bioaccumulation factors of examined crustose lichens showed their propensity to hyperaccumulate heavy metals. Contrarily, concentrations of metals in fruticose thalli of *S. nanodes* were, as a rule, lower than in the corresponding substrates. This indicates that the growth form of thalli and degree of thallus adhesion to the substrate has a significant impact on metal concentrations in lichens colonising post-smelting wastes. Nonlinear regression models described by power functions show that at greater levels of Pb concentration in the substrate, the ability of *C. aurella*, *L. muralis* and *L. fuscoatra* to accumulate the metal experiences a relative decrease, whereas hyperbolic function describes a similar trend in relation to Ni content in *S. nanodes*. This phenomenon may be an important attribute of lichens that facilitates their colonisation of the surface of slag wastes.

**Electronic supplementary material:**

The online version of this article (doi:10.1007/s00244-015-0180-5) contains supplementary material, which is available to authorized users.

The smelting of metal ores has resulted in large quantities of wastes deposited as slag dumps, which have artificial nature and contain high concentrations of heavy metals (e.g., Maciak 1996; Tyszka et al. 2014). In many heavy-metal-polluted sites, assemblages of cryptogams are the main visual components (Cuny et al. 2004a; Rola et al. 2014, 2015; Rola and Osyczka 2014). Lichens are known to be stress tolerators (Grime 1979), and some of them are well adapted to metal contamination (Purvis and Halls 1996; Cuny et al. 2004a). These symbiotic organisms appear to be effective and rapid colonisers of bare substrates including extremely contaminated slag dumps (Osyczka and Rola 2013a). Heavy metal accumulation capacity of lichens growing directly on the surface of slag sinters raises important questions about the colonisation abilities and metal tolerances of particular species. Some lichens appear to be specific to metal-rich substrates, and most of them belong to crustose genera such as *Acarospora*, *Aspicilia*, *Candelariella*, *Lecanora*, *Lecidea*, *Porpidia*, *Rhizocarpon*, or *Tremolecia* (Nash III 1989; Purvis and Halls 1996). Some species show a great ability to accumulate exceptionally high amounts of metals and thus are frequently considered as hyperaccumulators: for instance, *Diploschistes muscorum* (Sarret et al. 1998; Cuny et al. 2004b), *Acarospora rugulosa* (Chisholm et al. 1987) and *Lecanora polytropa* (Pawlik-Skowrońska et al. 2006).

Lichens bioaccumulate both essential and nonessential elements through various mechanisms including surface complexation, biomineralisation, and physical trapping of dust and soil particulates in the intercellular spaces of the medulla (Richardson 1995; Wilson 1995; Nash III 2008a). The whole surface of the thallus is involved in the absorption, so that elements present in the atmosphere as well as those present in the substrate can penetrate into the lichens’ bodies (Tyler 1989; Basile et al. 2008; Nash III 2008a). Because lichens lack root systems, it is widely believed that atmospheric deposition is the main source of elements in the thalli. However, if the concentrations of elements in the substrate are much greater than the deposition of elements from the atmosphere, accumulation in lichen thalli could be substrate-dependent (de Bruin and Hackenitz 1986; Loppi et al. 1999; Bajpai et al. 2009). Thus, lichens growing on metal-enriched soils accumulate large amounts of elements that reflect variations in the chemical properties of the substrate (Nash III 1989). Metal accumulation could largely depend on the substrate type and habitat requirements of a particular species. Some investigators, having recorded a weak correlation between lichen and substrate chemistry, have suggested that the substrate is not a main source of metals (Chiarenzelli et al. 1997; Bajpai and Upreti 2012). In contrast, others have confirmed metal ion uptake from a mineral substrate and noted that element concentrations in lichens were parallel to those in the underlying rock (Bargagli et al. 1987; Chettri et al. 1997; Bačkor and Fahselt 2004). The growth form of a lichen is also an important factor affecting the acquisition of elements (Chiarenzelli et al. 1997; Bačkor and Loppi 2009). Nevertheless, information is still needed on metal content in lichens in relation to metal concentrations in the substrates on which they grow.

Metal accumulation studies of epilithic lichens are limited and often difficult to perform due to lichens’ relatively low biomass and intimate association with the substrate (see Bačkor and Fahselt 2004). The aim of the present study was to investigate the behaviour of epilithic lichens, representing two main types of thallus morphology, in terms of bioaccumulation capacities and accumulation patterns as their adaptation to such extreme conditions. We determined the relationships between element content in the thalli and the corresponding substrates. We set the following hypotheses: (1) the substrate is the main source of heavy metals in the thalli of epilithic lichens; furthermore, the content of elements in the thallus correlates with that in the corresponding substrate; (2) growth form plays a significant role in the accumulation of metal by lichens; and (3) crustose lichens possess the ability to hyperaccumulate heavy metals.

## Materials and Methods

### Study Area

The study is based on material collected from three post-smelting dumps located within the Upper Silesian Industrial Region, southern Poland. The centres of the dumps are 50°15′58″N, 18°52′11″E; 50°16′42″N, 18°51′51″E; and 50°21′11″N, 18°58′00″E, and the elevation ranges from 260 to 291 m a.s.l. (Fig. S1). The dumps share the same origin and were deposited as a result of the processing of zinc–lead ores in adjacent smelters. They are examples of disturbed environments with high concentrations of toxic elements and unfavourable habitat factors (Skubała 2011, 2014; Osyczka and Rola 2013a; Rola et al. 2015). Heavy metals are among the most numerous groups of elements in the chemical composition of metallurgical slag (Jończy 2012; Tyszka et al. 2014). Other wastes, comprising the remnants of disused and demolished units of smelters and blast furnaces such as fragments of crucibles and bricks, concrete and asbestos elements, also occur. Concentrations of heavy metals in the material of dumps often significantly exceed permissible levels even for post-industrial areas (see Kabata-Pendias and Pendias 2001; Osyczka and Rola 2013a; Rola et al. 2015). Nevertheless, the wastes are inhomogeneous in terms of element concentrations owing to the long duration of their deposition during which different ores were used (Puziewicz et al. 2007; Tyszka et al. 2014). This gave us an opportunity to study a wide spectrum of particular element concentrations in substrates.

### Materials and Sampling

Four lichens were selected for this study: *Candelariella aurella* (Hoffm.) Zahlbr., *Lecanora muralis (Schreb.) Rabenh., Lecidea fuscoatra* (L.) Ach. and *Stereocaulon nanodes* Tuck. (Fig. S1A–D). These species are generally epilithic and frequently grow on manmade and metal-enriched basic substrates in urban areas (Purvis and Halls 1996; Smith et al. 2009). They are sturdy colonisers of post-smelting dumps and are numbered among the very few lichens able to grow directly on slag sinters (Osyczka and Rola 2013a). *C. aurella* and *L. fuscoatra* are typically crustose lichens strictly adhering to their substrates. *L. muralis* is also commonly classified as a crustose lichen; however, its thallus consists of placodioid circular patches or rosettes. *S. nanodes* forms fine fruticose secondary thalli which protrude from the ground. Lichen specimens were determined under stereoscopic and light microscopes; lichen secondary compounds, required for the precise determination of certain species, were analysed by means of thin layer chromatography (TLC) according to Orange et al. (2001).

Two types of substrate were considered, i.e., diversified solid slag sinters and concrete/asbestos-cement wastes (treated as a reference material) deposited on the dumps. Thirty specimens of each lichen species, together with the host substrates, were collected for chemical analysis; 24 specimens relate to artificial slag sinters and 6 to concrete/asbestos-cement elements. Collected lichens were represented by individuals in generally good life condition and with similar stage of thalli development. All specimens for a single species were collected from one area of a post-smelting dump in a short period of time (2013, summer season) under mostly uniform weather conditions (see Fig. S2). Our sampling strategy was designed so as to achieve possibly the wide spectrum of metal enrichment in the host substrates of particular lichen species and eliminate, or at least decrease, the air pollution factor as well as other external factors, e.g., weather conditions, seasonal differences, microclimate, pH (see also Goyal and Seaward 1981; Haas et al. 1998; Ferretti and Erhardt 2002; Shukla et al. 2014)..

### Element Determination

Total concentrations of zinc (Zn), lead (Pb), cadmium (Cd), and nickel (Ni) were determined in the lichen and substrate samples. These elements were chosen because they represent major contaminants of studied smelting slags (Jończy 2012; Tyszka et al. 2014) and, in excess, may induce toxic effect on lichens (Nash III 2008b). Small substrate fragments obtained from beneath the lichen thalli were first crushed in a ceramic mortar and then ground to a powder in an agate grinder. Approximately 20 mg of dry substrate material was digested in a solution of 65 % HNO_3_ (Suprapur, Merck) and 70 % HClO_4_ (Suprapur, Merck) (2:1 ratio). Each lichen sample constituted the thallus precisely separated from a single fragment of host substrate with stainless steel scalpels and forceps under a stereoscopic microscope. Macroscopic foreign materials adhering to thalli surfaces were carefully removed. In addition, lichen samples were once rinsed with deionised water to remove dust particles of weathered slag adhering to the surface of thalli, dried at 90° C for approximately 24 h to a constant weight, and then ground into powder (Pawlik-Skowrońska and Bačkor 2011; Vantová et al. 2013). Approximately 10 mg of dry lichen material was digested in 65 % HNO_3_ (Suprapur, Merck) and 30 % H_2_O_2_ (2:1 ratio). The samples were diluted with double-distilled water. Concentrations of particular elements in the substrate and lichen samples were determined using a Unicam Solar 939 atomic absorption spectrometer (Unicam Limited, Cambridge, UK) and the air-acetylene flame method. Certified standard solutions (Merck-Titrisol) were used to prepare the elemental calibration standards and for quality assurance. Appropriate solutions without samples were used as reagent blanks. Analyses of elements were repeated three times for each digest, and the mean values were used as one observation. The values of relative standard deviation (RSD) for Zn, Cd, Pb and Ni were within the ranges 0.0–8.5, 0.2–14.1, 0.0–9.7, and 0.1–10.9 %, respectively. In addition, reliability of results was checked by analysing the reference materials (INCT-PVTL-6, SRM-1577b); the recovery ranged from 97 to 111 %. Detection limit values of elements (mg/L) were as follow: 0.013 for Zn, 0.10 for Pb, 0.032 for Cd, and 0.063 for Ni.

### Statistical Analysis

Statistical calculations were performed with STATISTICA 10. The differences between metal concentrations in slag and reference substrates were tested by means of Student *t* test. Before the analysis, the variables were Box–Cox-transformed to find the optimal normalising transformation for each variable. Significant effects of lichen species and substrate type on particular element concentrations in the lichen thallus were calculated by means of multivariate analysis of variance (MANOVA) using Wilk’s lambda test statistic. The dependent variables were tested for homogeneity of variance using Levene’s test. When the result of MANOVA was significant (*p* < 0.05), univariate tests for each element and Tukey’s honest significant difference (HSD) post hoc tests were performed to detect significant differences between particular species and substrate types. The bioaccumulation factors of particular species were calculated according to the formula: BAF = concentration of element in the lichen/concentration of element in the corresponding substrate.

For initial evaluation of the relations between Zn, Pb, Cd and Ni concentrations in lichen thalli and in the corresponding substrates, scatterplots were created. Afterward, the distribution normality of each variable was verified using the Lilliefors test, and Pearson or Spearman correlation coefficients were calculated. Subsequently, detailed regression analysis was performed only in the case of homogenous groups with continuous data in which significant correlations were detected. Various regression models, i.e., linear regression and curvilinear regression models described by power, exponential, semilog, hyperbolic and polynomial functions, were considered based on evaluation of scatterplots. For this purpose, the variables were appropriately transformed to convert curvilinear models into linear models, in which parameters could be determined by least squares estimation (Sen and Srivastava 1990). The best-fitting model was chosen for further consideration (see Motulsky and Christopoulos 2004) according to the coefficient of determination (*R*^2^). A detailed residual analysis was performed to obtain reliable regression coefficients and to detect outliers. Formulas obtained for linearised regression models were transformed to the final form of the appropriate function.

## Results

### Heavy Metal Contents in Substrates

**Fig. 1:**
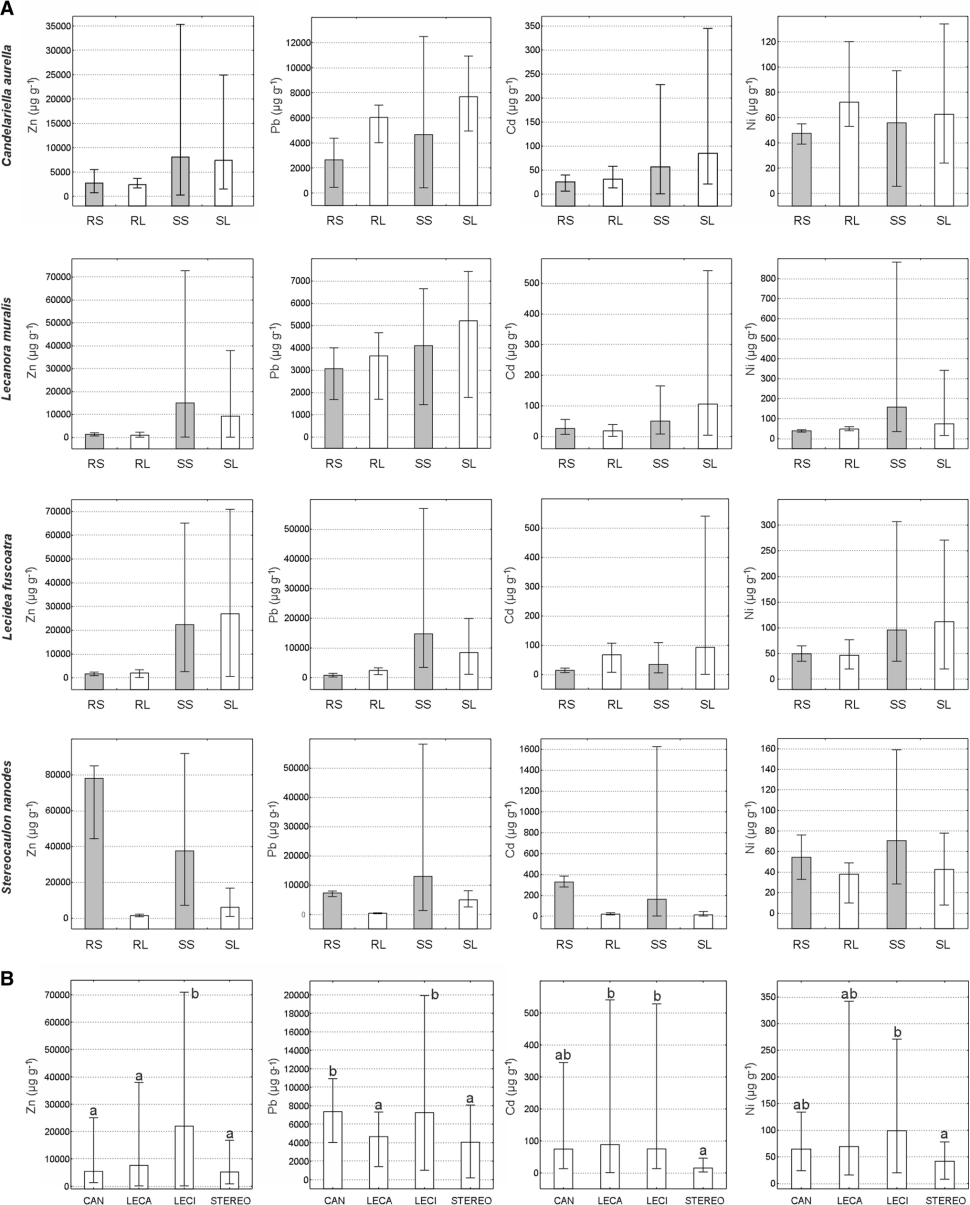
**a** Concentrations of particular elements in substrates (*grey bars*) and lichen thalli (*white bars*) across all studied species. **b** Overall concentration of elements in the thalli of particular lichens. *Bars* indicate mean values; *whiskers* show minimum and maximum values. RS reference substrate, *RL* lichen thalli collected from reference substrate, *SS* slag substrate, *SL* lichen thalli collected from slag substrate, *CAN C. aurella*, *LECA L. muralis*, *LECI L. fuscoatra*, *STEREO S. nanodes*.* Letters* denote results of Tukey’s HSD test;* different letters* indicate significant differences at the *p* < 0.05 level

A host substrate colonised by studied lichens shows a wide spectrum of heavy metal contents; the ranges were as follows: Zn = 0.02–9.27 %, Pb = 0.02–5.82 %, Cd = 0.61–1625 µg g^−1^, and Ni = 5.65–883 µg g^−1^. Wide fluctuations in the concentration of metals, in particular in the case of sinter slag (Fig. 1 and Supplementary Information Table S1), indicate that the wastes deposited in dumps may vary considerably in terms of composition and potential toxicity. Nevertheless, slag was usually characterised by highly increased concentrations of heavy metals reaching enormous maximum levels unparalleled by natural solid surfaces inhabited by lichens. Slag substrates usually contained more heavy metals than reference substrates. However, concrete/asbestos rubble, due to its origin, was also often heavily contaminated (Fig. 1). Therefore, significant differences (*p* < 0.05) were found only between concentrations of Pb and Ni in slag and reference substrates.

### Heavy Metal Accumulation in Lichens

**Fig. 2:**
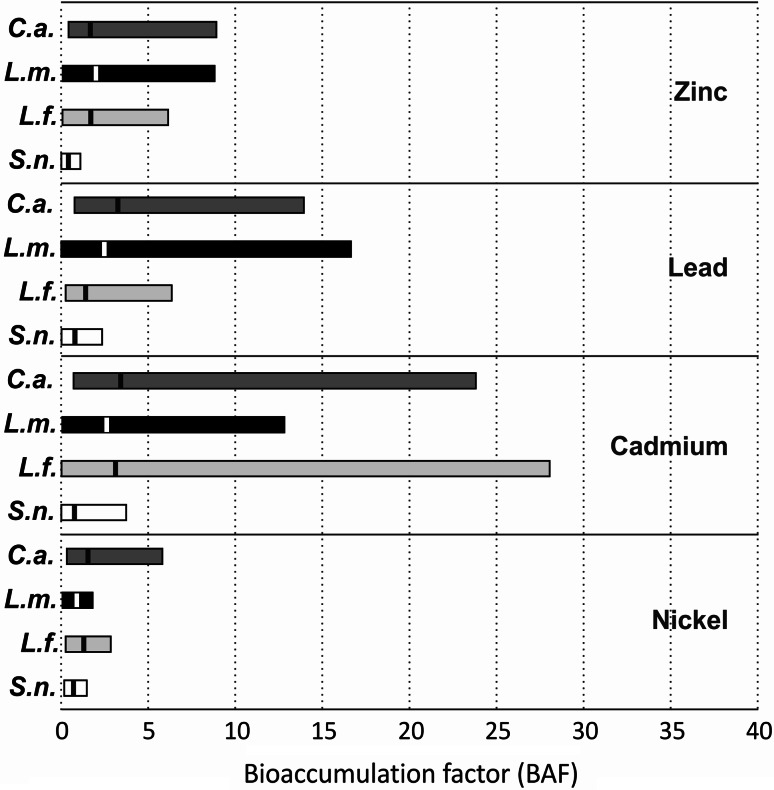
Ranges of BAFs calculated as the ratio of metal content in a lichen thallus and the corresponding substrate. Mean values are indicated by *vertical lines* within the *bars*. *C.a. C. aurella*, *L.m.* *L. muralis,* *L.f. L. fuscoatra*, *S.n.* *S. nanodes*

The comparison of BAF between studied species shown that the accumulation capacities of crustose lichens were much greater than those of *S. nanodes* in relation to all examined elements (Fig. 2). Calculated mean values of BAF exceeded 1 for all crustose lichens with only one exception: the bioaccumulation factor of Ni in *L. muralis* was slightly lower. In contrast, concentrations of metals in the fruticose thalli of *S. nanodes* were as a rule lower than in the corresponding substrates. Moreover, the concentration levels of heavy metals in many crustose lichen samples were several times greater than in their host substrates: 2149 versus 253 μg g^−1^ of Zn, 31 versus 6 μg g^−1^of Ni for *C. aurella*, 4427 versus 266 μg g^−1^of Pb for *L. muralis*, and 156 versus 6 μg g^−1^of Cd for *L. fuscoatra*. The content of Pb and Zn reached as high as several percent of the dry weight (dw) of some crustose lichen samples (Fig. 1). The maximum Zn and Pb burdens were observed in *L. fuscoatra* (as much as approximately 7.1 % and approximately 1.9 % dw of thallus, respectively), and the highest concentrations of Cd and Ni were detected in *L. muralis* (541 and 342 μg g^−1^, respectively).

MANOVA showed a highly significant main effect of species on the concentration of metal elements in lichen thallus (Wilk’s lambda = 0.715; *p* = 0.000) (Table 1). Subsequent univariate tests for selected elements showed significant differences for species only in the case of Pb concentration. The type of substrate also had an important effect on metal concentrations in lichens (Wilk’s lambda = 0.768; *p* = 0.000). Significant differences (*p* < 0.05) were recorded regarding the content of Zn and Pb in lichen thalli between the samples collected from slags and concrete elements. Furthermore, a highly significant species × substrate interaction effect (Wilk’s lambda = 0.753; *p* = 0.002) was observed regarding Pb.

**Table 1:** Results of MANOVA to determine the effects of lichen species and substrate type on the concentrations of particular elements in lichen thalli

Element	Species^a^		Substrate^b^		Species × Substrate	
	*F*	*p*	*F*	*p*	*F*	*p*
Zn	2.422	0.069	**13.110**	**0.000**	2.337	0.078
Pb	**8.285**	**0.000**	**29.495**	**0.000**	**3.688**	**0.014**
Cd	1.438	0.236	3.651	0.059	1.053	0.372
Ni	1.728	0.166	3.291	0.072	1.748	0.162

Bold values denote significant differences at the *p* < 0.05 level

^a^For studied species *C. aurella*, *L. muralis*, *L. fuscoatra*, and *S. nanodes*

^b^For two types of substrate: slag and concrete

### Relationships Between Heavy Metal Content in Lichens and Substrate

**Fig. 3:**
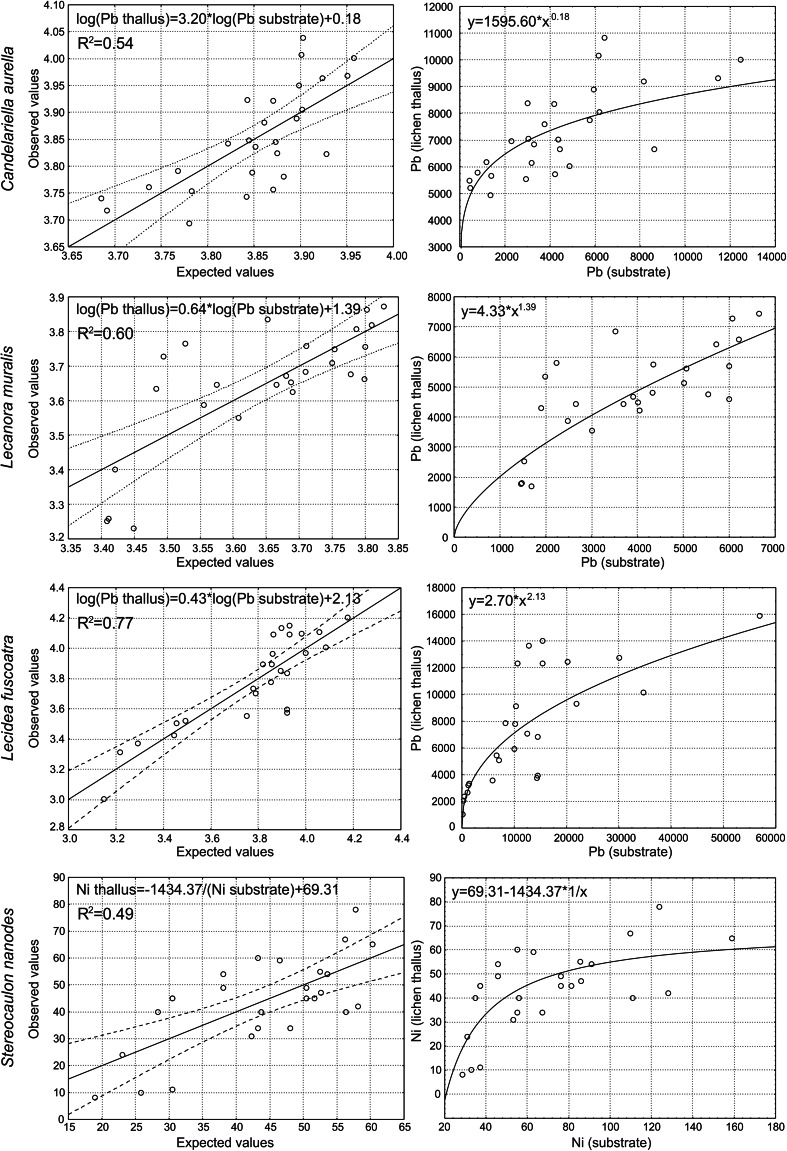
Linearised regression plots of observed versus expected values of dependent variables together with scatterplots including fitted functions presenting the relationships between concentrations of elements in lichen thalli and the corresponding substrates for particular species

**Fig. 4:**
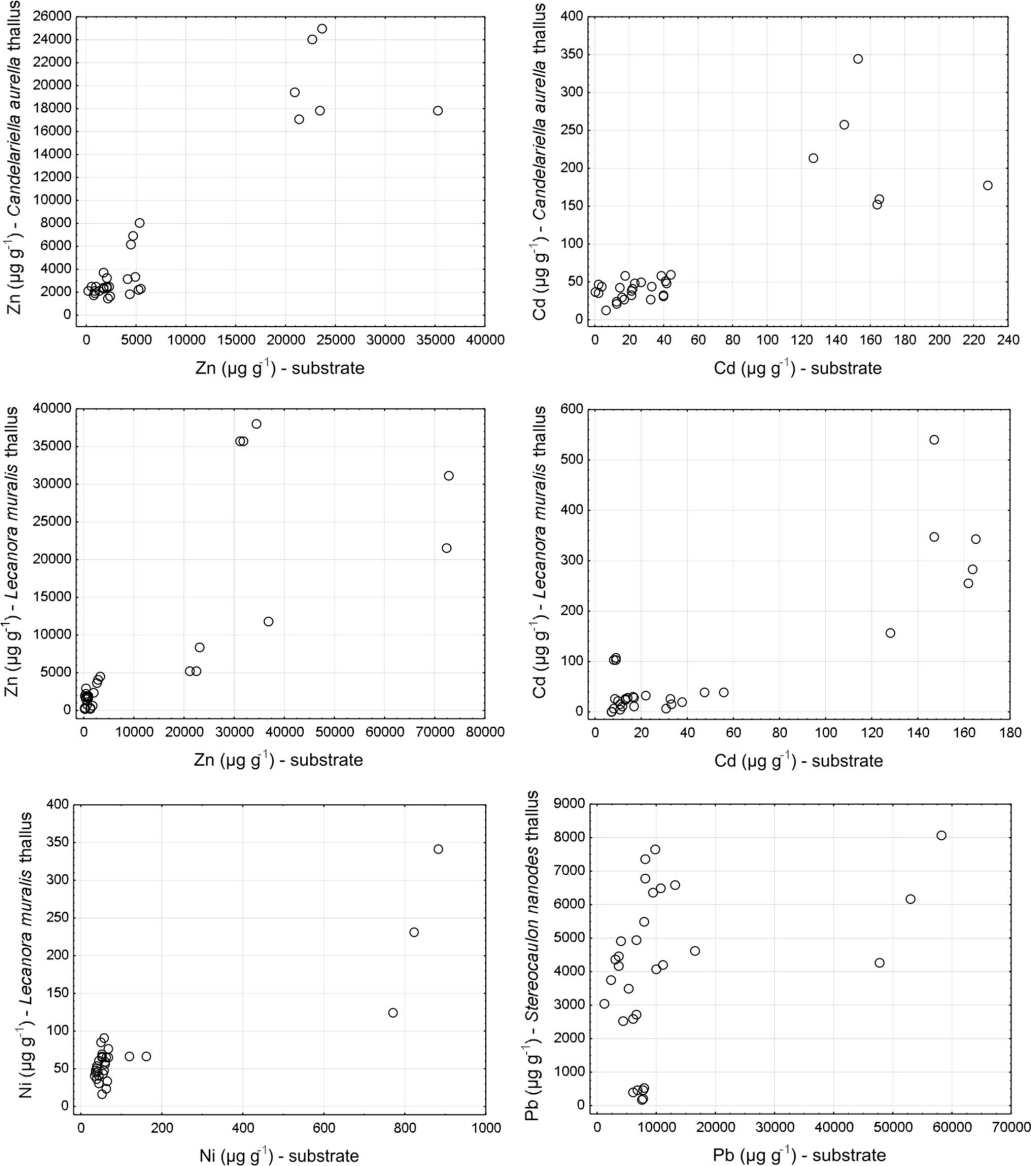
Scatterplots presenting the relationships between the concentrations of particular metal elements in lichen thalli and the corresponding substrates for studied species in which significant (*p* < 0.05) Spearman correlation coefficients were found

Several significant correlations between the concentrations of metal elements in the lichen thalli and corresponding substrate were found (Table 2). The preliminary analysis of scatterplots of these relationships suggested that at least in some cases they are certainly nonlinear. Consequently, the specific regression models reflecting the Pb content relationships for *C. aurella*, *L. muralis*, and *L. fuscoatra*, as well as for Ni content in the case of *S. nanodes*, were defined (Fig. 3). Power functions proved to be the best fits for the empirical data, explaining 54.2, 60.2, and 77.0 % of the variation for the first three relations concerning Pb content, whereas hyperbolic function was the most appropriate to describe the relationship between the content of Ni in the thalli of *S. nanodes* and the corresponding substrates. This means that along with an increasing content of heavy metals in the substrate, the accumulation capacity of the above-mentioned species in relation to these elements decreases approximately in accordance with the designated functions. The data did not allow definition of significant models in the case of other relationships; however, certain tendencies can be observed. The scatterplots (Fig. 4) show that at greater concentrations of elements in substrates, the contents of these elements in lichen thalli are not high in relation to the rapid increase observed at low concentrations. This may suggest that in these cases that accumulation is also limited and that increases in metal concentrations in substrates do not automatically cause linear increases in their concentrations in lichen thalli.

**Table 2:** Pearson or Spearman correlation coefficients between the concentrations of heavy metals in lichen thalli and the corresponding substrates for particular species

Species	Element			
	Zn	Pb	Cd	Ni
*C. aurella*	***R***_**S**_ **=** **0.68**	***R*** **=** **0.52**	***R***_**S**_ **=** **0.70**	*R* = 0.10
*L. muralis*	***R***_**S**_ **=** **0.81**	***R*** **=** **0.54**	***R***_**S**_ **=** **0.59**	***R***_**S**_ **=** **0.51**
*L. fuscoatra*	*R* = 0.23	***R*** **=** **0.64**	*R*_S_ = 0.11	*R* = 0.36
*S. nanodes*	*R* = −0.05	***R***_**S**_ **=** **0.48**	*R*_S_ = 0.46	***R*** **=** **0.53**

Bold values denote significant correlations at the *p* < 0.05 level

*R* Pearson correlation coefficient, *R*_S_ Spearman correlation coefficient

## Discussion

### Metal Accumulation Capacity of Epilithic Lichens

The studied species have frequently been noted on metal-rich substrates (e.g., Hickmott 1980; Ernst et al. 2004); however, their detailed accumulation capacities in relation to the heavy metals present in the substrate have not yet been investigated. They clearly have the potential to accumulate large amounts of heavy metals, especially those with a crustose growth form. This is evidenced by the ability to achieve high BAF (Fig. 2), and a high metal uptake was also documented in some previous papers; for example, *L. fuscoatra* from smelting slag heaps in Germany accumulated Pb at levels reaching as much as 4 % of dry matter (Lange and Ziegler 1963). Similarly, *L. muralis* proved to have a high capacity for Pb absorption (Tsigaridas et al. 2015), and *S. nanodes* was considered to possess an affinity for Pb and Zn (Noeske et al. 1970; Maquinay et al. 1961). Our study did not confirm exceptional accumulation capacity of *S. nanodes* for Zn and Pb compared with the other species. However, in one sample the content of Zn was greater than in the substrate, and nearly 20 % of samples accumulated slightly greater amounts of Pb than was present in their host substrates.

Reports of high metal contents in lichen hyperaccumulators are mainly due to the trapping of particulate matter and extracellular accumulation (Purvis and Pawlik-Skowrońska 2008; Bačkor and Loppi 2009). Functional groups of mycobiont cell walls (Sarret et al. 1998), metal oxalates (Purvis 1984; Chisholm et al. 1987), and metal-lichenic acid complexes (Pawlik-Skowrońska et al. 2006; Pawlik-Skowrońska and Bačkor 2011) may be involved in extracellular metal accumulation. Epilithic lichens are known to encrust themselves onto rocks by exuding compounds that weather the rock-forming minerals by complexing their cations (e.g., Adamo and Violante 2000). Considering the high accumulation capacity of the studied crustose lichens, we can suppose that these species also possess certain physiological resistance. For example, the calcium oxalate produced by *L. fuscoatra* probably contributes to the extracellular binding of various metal cations (Vingiani et al. 2013) as was determined in the well-known hyperaccumulator *D. muscorum* (Sarret et al. 1998). Some secondary lichen metabolites, including usnic acid present in *L. muralis*, may function as extracellular heavy metal chelators on the surface of mycobiont structures (Purvis and Pawlik-Skowrońska 2008). Moreover, in view of the fact that dump materials emit great amounts of dust, we can suppose that entrapment of metal-rich particles originating from slag is largely responsible for high metal loadings in the studied lichens as has been suggested by several investigators (e.g., Nieboer et al. 1976; Nash III 2008b).

### Interaction Between Lichen Growth Form and Accumulation Capacity

The relationship between thallus morphology and the acquisition of metal elements is still poorly understood; the results of previous studies are rather inconclusive (see also Bačkor and Loppi 2009). Several investigators have observed a relation between the growth form and the accumulation capacity of particular species and concluded that it is a key factor affecting patterns of element accumulation in lichens (e.g., Chettri et al. 1997; Chiarenzelli et al. 1997; St. Clair et al. 2002). Our results are generally consistent with reports indicating that the crustose type of thallus is the most efficient accumulator of heavy metals (Chisholm et al. 1987; Sarret et al. 1998; Pawlik-Skowrońska et al. 2006). Contrarily, Bajpai et al. (2009) found that foliose and leprose thalli absorb greater amounts of arsenic than squamulose and crustose, whereas Pawlik-Skowrońska et al. (2008) found that contents of Zn and Pb varied widely among quite different lichens and suggested that the contents of these elements are independent of thallus morphology. Nevertheless, there is a great deal of evidence of high interspecific differences in metal accumulation (e.g., Nimis et al. 2001). Based on the data on the accumulation ability of fruticose lichen *Cladonia rei* colonising friable slag substrate, it can be noticed that its BAF were considerably lower than those determined for crustose lichens studied here (Fig. 2 cf., Osyczka and Rola 2013b). The intimate association of epilithic lichen with the slag, as well as the relatively great surface of their thalli directly adjacent to the substrate, are undoubtedly important factors responsible for greater accumulation of heavy metals.

### Restrained Accumulation Pattern of Epilithic Lichens

Heavy metal content in lichen thalli being greater than that that in the substrate is often explained by the atmospheric origin of the elements (e.g., Bajpai and Upreti 2012). Our results clearly indicate that the substrate is the main or at least a significant source of metals in the studied lichen samples (Table 2; Fig. 3), regardless of element-uptake methods. The fine slag particles may penetrate into the thalli directly from beneath the lichen or indirectly through the dust carried in the air. Although dust particles are dispersed in the dumps’ area by air, the source of metals in the thalli is still “soil-borne,” i.e., originating from substrate contamination. Similarly, Pawlik-Skowrońska et al. (2008) observed that lichens sampled in forests, in sandy areas, contained less metal than those living on heavy–metal–contaminated substrates of mine tailing dumps and that the content of metals in lichens reflected their content in the corresponding substrates. It is important to ask not only to what extent lichens accumulate metals and their potential sources in the thalli but also to determine the nature of this accumulation. A recent study showed that specific nonlinear regression models described by a power function most reliably reflected relationships between Zn and Cd contents in *Cladonia rei* thalli and in the host substrates. Such restrained accumulation pattern means that with increasing contents of Zn and Cd in the substrate, the accumulation capacity of this species in relation to these elements decreases (Osyczka and Rola 2013b). The present study showed that nonlinear relationships can also be observed in relation to some elements in epilithic species. We found that the studied crustose lichens resisted the excessive accumulation of Pb (Fig. 3). The content of metals in lichen thalli increases rapidly along with increased metal content in the corresponding substrates, but it does so only at the lower range of concentration of the element in the substrates (Figs. 3, 4). In the context of a wide range of substrate contamination, it can be clearly seen that at the greater range of metal concentration in the substrates, the capacity of lichens to accumulate the metal experiences a relative decrease. Contrarily to epilithic crustose lichens, in the case of epigeic fruticose *C. rei*, which also colonizes the same slag dumps, any correlation between Pb content in the thalli and substrate was observed as was its restrained accumulation pattern relating to Zn and Cd (Osyczka and Rola 2013b). In contrast, epilithic fruticose *S. nanodes* resists the excessive accumulation of Ni (Fig. 3). This showed that lichens with different ecology and growth forms differ in terms of their response to particular contaminants. This also indicates a potentially different main source of the elements in the thalli (see Loppi et al. 1999; Osyczka and Rola 2013b). Nevertheless, all defined models show a similar character and indicate that lichens species may show resistance to the increased accumulation of some heavy metals.

There are three main mechanisms of metal accumulation in lichen thalli: (1) trapping of solid particles; (2) extracellular binding with exchange sites on the cell walls of symbionts; and (3) intracellular uptake (Garty et al. 1979; Nash III 2008b). The contribution of individual mechanisms in the examined epilithic species could play a crucial role in explaining the model of restrained accumulation pattern showed in this study. Previous studies have shown that intracellular metal content in lichen thalli is relatively stable over time (Mikhailova and Sharunova 2008); however, laboratory investigations have showed that intracellular uptake of Cd can be decreased in the foliose thalli of *Peltigera* collected from contaminated localities compared with those collected from background sites (Beckett and Brown 1984a). Therefore, at high concentrations of toxic elements in the substrate, the metabolism of the examined lichens could be impaired and their biological accumulation decreased, even though extracellular passive binding of metals and particulate trapping would still occur (see, e.g., Beckett and Brown 1984b; Bačkor and Loppi 2009). It should be also mentioned that various elements are frequently distributed differently within a lichen thallus (Cuny et al. 2004b; Bačkor et al. 2011). Accordingly, loads, distribution, and deposition of metals within the thallus may depend on many factors such as thallus morphology, production of secondary metabolites, and specific properties of the species (see Bačkor and Loppi 2009).

## Conclusions

First, the growth form of lichens colonising post-smelting wastes has a significant impact on metal concentrations in the thalli. Crustose lichens accumulate considerably greater amounts of heavy metals than do fruticose lichens. The intimate association of epilithic lichen with the slag and relatively great surface of their thalli directly adjacent to the substrate are undoubtedly important factors responsible for their greater accumulation of heavy metals. Second, BAFs of crustose lichens are much greater than those of fruticose *S. nanodes*. The levels of heavy metals in crustose lichens, compared with fruticose lichens, were usually greater than those in their host substrate. Third, only the concentration of Pb in all studied epilithic lichens depends on both the element content in the substrate and the substrate type. However, the accumulation pattern of Pb differs between species. Fourth, lichens may resist the excessive accumulation of heavy metals. The relationships between element content in lichen thalli and the corresponding substrates are mostly nonlinear dependencies; along with increasing metal concentration in the substrates, the capacity of lichens for accumulation experiences a relative decrease.

## Electronic Supplementary Material

Supplementary material 1 (DOC 1609 kb)
