# Supplementary material for: Different Heavy Metal Accumulation Strategies of Epilithic Lichens Colonising Artificial Post-Smelting Wastes
Source: Arch Environ Contam Toxicol. 2015 Jul 9;70:418–28. doi: 10.1007/s00244-015-0180-5 (PMC4722062; doi:10.1007/s00244-015-0180-5)
Supplement: Supplementary file 1 — Supplementary material 1 (DOC 1609 kb) [file 244_2015_180_MOESM1_ESM.doc]

**Different heavy metal accumulation strategies of epilithic lichens colonising artificial post-smelting wastes**

**Kaja Rola ^a^, Piotr Osyczka ^b^*, Alina Kafel^c^**

^a^Department of Plant Taxonomy, Phytogeography and Herbarium, Institute of Botany, Jagiellonian University, Kopernika 27, 31-501 Kraków, Poland

^b^Department of Polar Research and Documentation, Institute of Botany, Jagiellonian University, Kopernika 27, 31-501 Kraków, Poland

^c^Department of Ecotoxicology and Animal Physiology, Faculty of Biology and Environmental Protection, University of Silesia, Bankowa 9, 40-007 Katowice, Poland

*Corresponding author. Tel.: +48 12 663 36 47

E-mail address: piotr.osyczka@uj.edu.pl (P. Osyczka)

**Fig. S1** Post-smelting slag dump deposited in Ruda Śląska town (Upper Silesian Industrial Region, southern Poland) and studied lichen species growing on slag sinters: A – *Candelariella aurella* (scale 5 mm), B – *Lecanora muralis* (scale 5 mm), C – *Lecidea fuscoatra* (scale 3 cm), D – *Stereocaulon nanodes* (scale 1 cm)


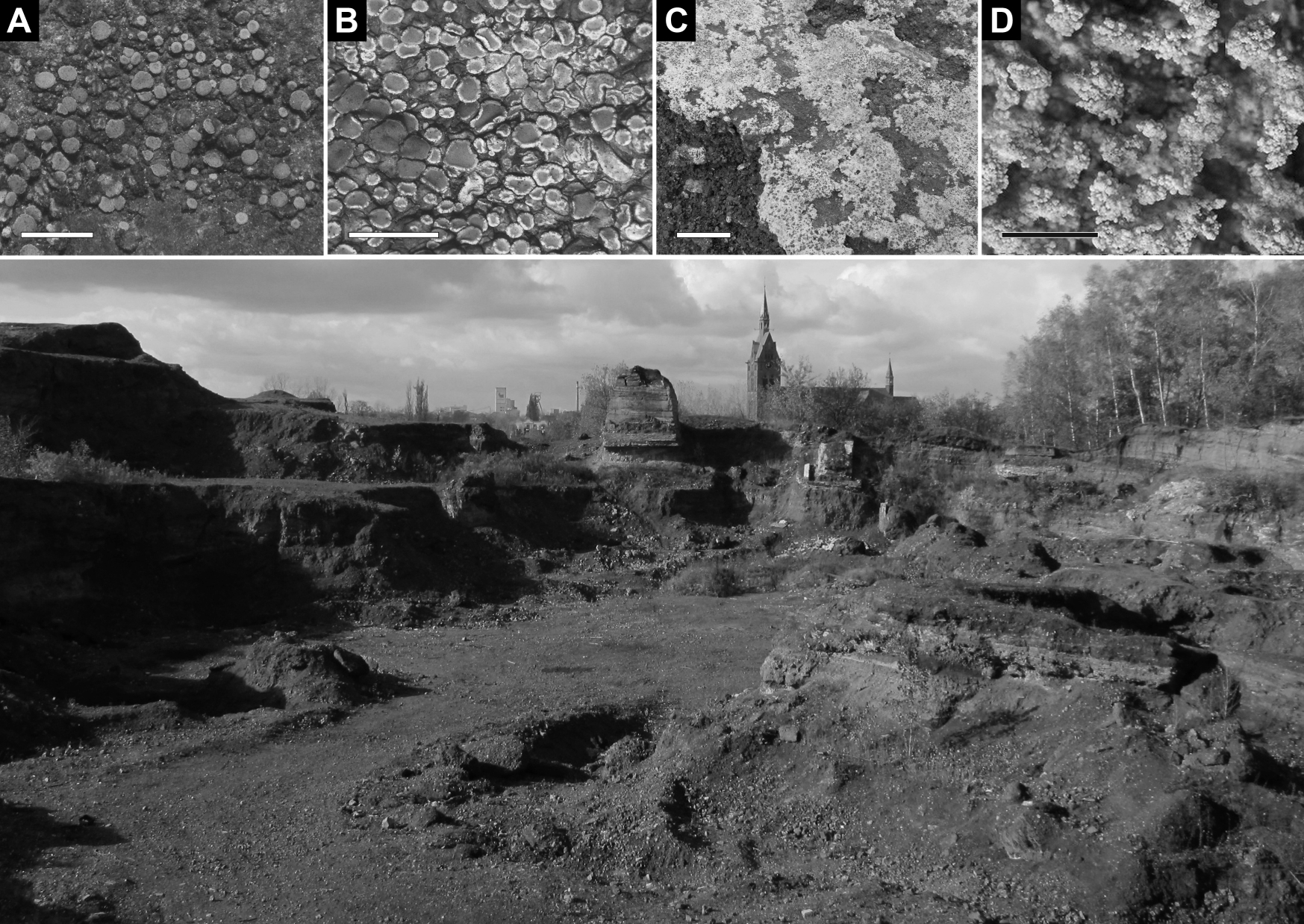


**Fig. S2** Ombrothermic diagram of the study area during the sampling period (prepared on the basis of data obtained from the nearest meteorological station, WMO index 12560)


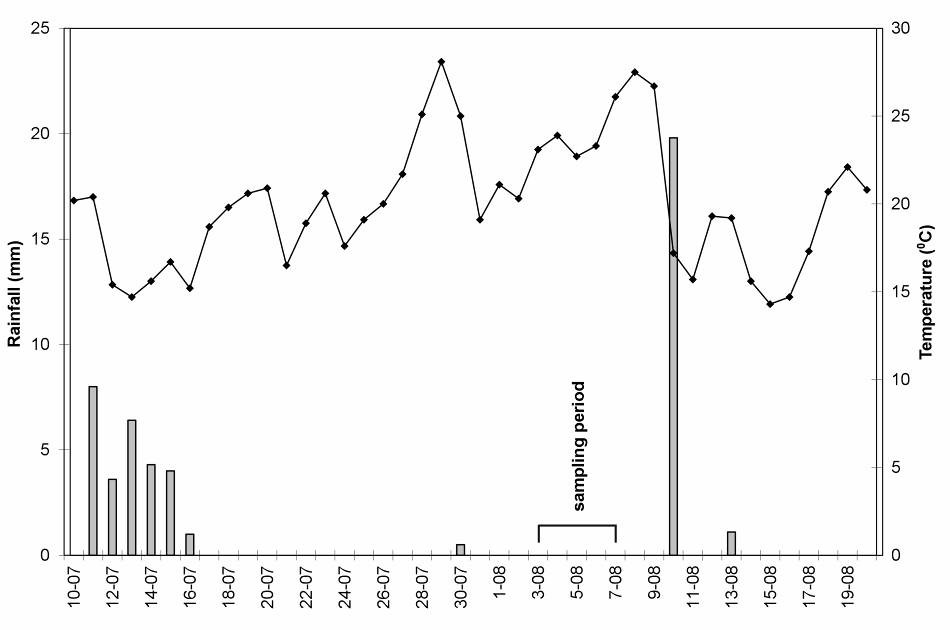


**Table S1** Overall mean value of element concentrations (± SD) in the thalli of lichen species (dry matter) collected from all slag sinters/reference materials along with concentrations of elements in corresponding substrates.

| Element | | Zn (μg g^–1^) | | Pb (μg g^–1^) | | Cd (μg g^–1^) | | Ni (μg g^–1^) | |
| --- | --- | --- | --- | --- | --- | --- | --- | --- | --- |
| Substrate | | slag | reference | slag | reference | slag | reference | slag | reference |
| Number of samples | | n=24 | n=6 | n=24 | n=6 | n=24 | n=6 | n=24 | n=6 |
| *Candelariella aurella* | Lichen | 7396.6±7872.7 | 2399.2±669.3 | 7681.5±1829.4 | 6029.2±1200.9 | 85.0±85.0 | 31.2±14.8 | 62.6±29.9 | 72.2±27.0 |
|  | Substrate | 8072.8±10147.8 | 2716.7±2085.7 | 4653.8±3174.8 | 2648.0±1326.2 | 56.7±66.3 | 25.5±14.0 | 55.8±23.9 | 47.5±6.0 |
| *Lecanora muralis* | Lichen | 9290.5±12672.5 | 981.5±881.0 | 5213.8±4015.5 | 3600.5±1410.0 | 105.7±143.5 | 18.4±18.0 | 74.5±69.8 | 48.7±7.8 |
|  | Substrate | 15053.3±22053.7 | 1353.5±543.2 | 4189.3±1824.2 | 3070.0±936.0 | 50.3±61.0 | 26.3±22.1 | 158.3±259.5 | 39.7±4.5 |
| *Lecidea fuscoatra* | Lichen | 26963.5±24352.0 | 2027.0±1133.2 | 8451.6±4965.4 | 2431.3±846.9 | 92.9±147.8 | 67.4±42.4 | 112.0±87.0 | 46.7±23.0 |
|  | Substrate | 22407.2±17664.3 | 1668.8±507.1 | 14759.5±11814.3 | 809.0±514.1 | 34.4±28.3 | 14.2±5.4 | 95.9±53.4 | 49.5±11.0 |
| *Stereocaulon nanodes* | Lichen | 6126.3±3992.0 | 1480.0±678.5 | 4960.5±1633.5 | 371.2±142.9 | 13.5±9.2 | 20.9±7.7 | 42.6±20.0 | 38.0±14.6 |
|  | Substrate | 37569.8±26852.0 | 79033.3±18551.5 | 12973.4±15954.1 | 7307.8±704.7 | 163.4±423.3 | 328.4±35.3 | 70.6±34.5 | 54.5±20.9 |
